# *N*-(2,6-Dimethyl­phen­yl)succinimide

**DOI:** 10.1107/S160053680905555X

**Published:** 2010-01-09

**Authors:** B. S. Saraswathi, B. Thimme Gowda, Sabine Foro, Hartmut Fuess

**Affiliations:** a Department of Chemistry, Mangalore University, Mangalagangotri 574 199, Mangalore, India; b Institute of Materials Science, Darmstadt University of Technology, Petersenstrasse 23, D-64287 Darmstadt, Germany

## Abstract

The mol­ecule of the title compound, C_12_H_13_NO_2_, lies on a twofold rotation axis that passes through the N and C_para_ atoms as well as through the mid-point of the bond between the methyl­ene C atoms. The dihedral angle between the aromatic ring and the amide segment is 75.9 (1)°.

## Related literature

For our studies on the effect of ring and side-chain substitutions on the structures of this class of compounds, see: Gowda *et al.* (2007 ▶, 2009**a* ▶,b* ▶).
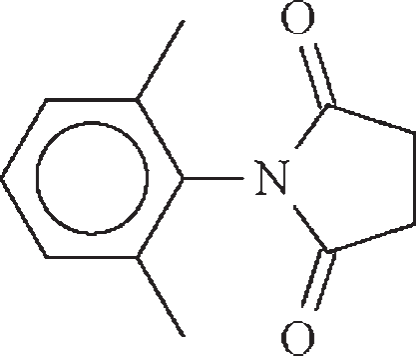


## Experimental

### 

#### Crystal data

C_12_H_13_NO_2_
*M* *_r_* = 203.23
Tetragonal, 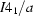

*a* = 9.4048 (3) Å
*c* = 23.685 (1) Å
*V* = 2094.94 (13) Å^3^
*Z* = 8
Mo *K*α radiation
μ = 0.09 mm^−1^
*T* = 299 K
0.44 × 0.44 × 0.40 mm

#### Data collection

Oxford Diffraction Xcalibur diffractometer
Absorption correction: multi-scan (*CrysAlis RED*; Oxford Diffraction, 2009 ▶)’ *T*_min_ = 0.962, *T*_max_ = 0.966
7329 measured reflections
1062 independent reflections
942 reflections with *I* > 2σ(*I*)
*R*_int_ = 0.024

#### Refinement

*R*[*F* ^2^ > 2σ(*F* ^2^)] = 0.058
*wR*(*F* ^2^) = 0.150
*S* = 1.11
1062 reflections
70 parameters
H-atom parameters constrained
Δρ_max_ = 0.21 e Å^−3^
Δρ_min_ = −0.45 e Å^−3^

### 

Data collection: *CrysAlis CCD* (Oxford Diffraction, 2009 ▶); cell refinement: *CrysAlis CCD*; data reduction: *CrysAlis RED* (Oxford Diffraction, 2009 ▶); program(s) used to solve structure: *SHELXS97* (Sheldrick, 2008 ▶); program(s) used to refine structure: *SHELXL97* (Sheldrick, 2008 ▶); molecular graphics: *PLATON* (Spek, 2009 ▶); software used to prepare material for publication: *SHELXL97*.

## Supplementary Material

Crystal structure: contains datablocks I, global. DOI: 10.1107/S160053680905555X/ng2713sup1.cif

Structure factors: contains datablocks I. DOI: 10.1107/S160053680905555X/ng2713Isup2.hkl

Additional supplementary materials:  crystallographic information; 3D view; checkCIF report

## Figures and Tables

**Fig. 1.**
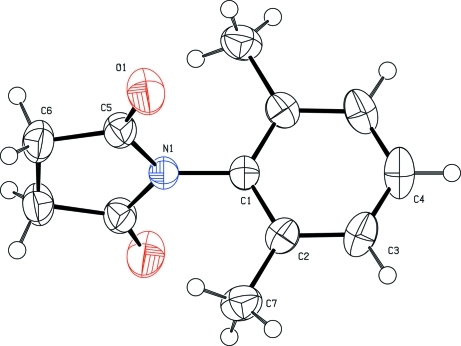
Molecular structure of the title compound, showing the atom labelling scheme. The displacement ellipsoids are drawn at the 50% probability level. The H atoms are represented as small spheres of arbitrary radii.

**Fig. 2.**
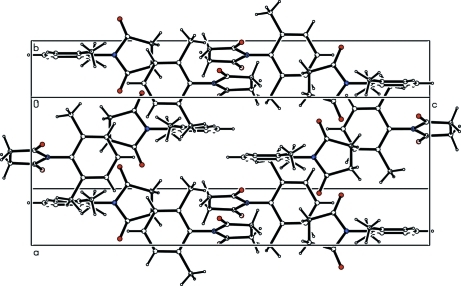
Molecular packing of the title compound.

## supplementary crystallographic information

### Comment

The amide moiety is an important constituent of many biologically significant compounds. As a part of studying the effect of ring and side chain substitutions on the structures of this class of compounds (Gowda *et al.*, 2007; 2009*a,b*), the crystal structure of *N*,*N*-(2,6-dimethylphenyl)succinimide has been determined (I) (Fig. 1).

The structure shows crystallographic inversion symmetry: there is one half-molecule in the asymmetric unit. The dihedral angle between the part of benzene ring and part of the amide segment in the two halves of the molecule is 75.9 (1)°.

The torsional angles of the groups, C2$1 - C1 - N1 - C5, C2 - C1 - N1 - C5, C2$1 - C1 - N1 - C5$1 and C2 - C1 - N1 - C5$1 in the molecule are -73.9 (1)°, 106.1 (1)°, 106.1 (1)° and -73.9 (1)°, respectively.

The packing of molecules into column like infinite chains parrallel to the *a*-axis is shown in Fig.2.

### Experimental

The solution of succinic anhydride (0.025 mole) in toluene (25 ml) was treated dropwise with the solution of 2,6-dimethylaniline (0.025 mole) also in toluene (20 ml) with constant stirring. The resulting mixture was stirred for one hour and set aside for an additional hour at room temperature for the completion of reaction. The mixture was then treated with dilute hydrochloric acid to remove the unreacted 2,6-dimethylaniline. The resultant solid *N*-(2,6-dimethylphenyl)succinamic acid was filtered under suction and washed thoroughly with water to remove the unreacted succinic anhydride and succinic acid. It was recrystallized to constant melting point from ethanol. *N*-(2,6-Dimethylphenyl)succinamic acid was then heated for 2 h and then allowed to cool slowly to room temperature to get crystals of *N*-(2,6-dimethylphenyl)succinimide. The purity of the compound was checked by elemental analysis and characterized by its infrared spectra. The prism like colourless single crystals of the compound used in X-ray diffraction studies were grown in ethanolic solution by slow evaporation at room temperature.

### Refinement

The H atoms were positioned with idealized geometry using a riding model with C—H = 0.93–0.97 Å. Isotropic displacement parameters for the H atoms were set equal to 1.2 *U*_eq_ (parent atom).

### Crystal data

**Table:**

C_12_H_13_NO_2_	*D*_x_ = 1.289 Mg m^−^^3^
*M**_r_* = 203.23	Mo *K*α radiation, λ = 0.71073 Å
Tetragonal, *I*4_1_/*a*	Cell parameters from 5744 reflections
Hall symbol: -I 4ad	θ = 2.8–27.9°
*a* = 9.4048 (3) Å	µ = 0.09 mm^−^^1^
*c* = 23.685 (1) Å	*T* = 299 K
*V* = 2094.94 (13) Å^3^	Prism, colourless
*Z* = 8	0.44 × 0.44 × 0.40 mm
*F*(000) = 864	

### Data collection

**Table:**

Oxford Diffraction Xcalibur diffractometer	1062 independent reflections
Radiation source: fine-focus sealed tube	942 reflections with *I* > 2σ(*I*)
graphite	*R*_int_ = 0.024
Rotation method data acquisition using ω and φ scans.	θ_max_ = 26.4°, θ_min_ = 3.4°
Absorption correction: multi-scan (*CrysAlis RED*; Oxford Diffraction, 2009)'	*h* = −11→11
*T*_min_ = 0.962, *T*_max_ = 0.966	*k* = −11→11
7329 measured reflections	*l* = −28→26

### Refinement

**Table:**

Refinement on *F*^2^	Primary atom site location: structure-invariant direct methods
Least-squares matrix: full	Secondary atom site location: difference Fourier map
*R*[*F*^2^ > 2σ(*F*^2^)] = 0.058	Hydrogen site location: inferred from neighbouring sites
*wR*(*F*^2^) = 0.150	H-atom parameters constrained
*S* = 1.11	*w* = 1/[σ^2^(*F*_o_^2^) + (0.0961*P*)^2^ + 0.6791*P*] where *P* = (*F*_o_^2^ + 2*F*_c_^2^)/3
1062 reflections	(Δ/σ)_max_ < 0.001
70 parameters	Δρ_max_ = 0.21 e Å^−^^3^
0 restraints	Δρ_min_ = −0.45 e Å^−^^3^

### Special details

**Table:**

Geometry. All e.s.d.'s (except the e.s.d. in the dihedral angle between two l.s. planes) are estimated using the full covariance matrix. The cell e.s.d.'s are taken into account individually in the estimation of e.s.d.'s in distances, angles and torsion angles; correlations between e.s.d.'s in cell parameters are only used when they are defined by crystal symmetry. An approximate (isotropic) treatment of cell e.s.d.'s is used for estimating e.s.d.'s involving l.s. planes.
Refinement. Refinement of *F*^2^ against ALL reflections. The weighted *R*-factor *wR* and goodness of fit *S* are based on *F*^2^, conventional *R*-factors *R* are based on *F*, with *F* set to zero for negative *F*^2^. The threshold expression of *F*^2^ > σ(*F*^2^) is used only for calculating *R*-factors(gt) *etc*. and is not relevant to the choice of reflections for refinement. *R*-factors based on *F*^2^ are statistically about twice as large as those based on *F*, and *R*- factors based on ALL data will be even larger.

### Fractional atomic coordinates and isotropic or equivalent isotropic displacement parameters (Å^2^)

**Table:**

	*x*	*y*	*z*	*U*_iso_*/*U*_eq_	
C1	0.5000	0.2500	0.10156 (7)	0.0325 (4)	
C2	0.38264 (14)	0.30632 (13)	0.13004 (6)	0.0376 (4)	
C3	0.38515 (18)	0.30545 (16)	0.18861 (6)	0.0499 (4)	
H3	0.3086	0.3426	0.2086	0.060*	
C4	0.5000	0.2500	0.21748 (9)	0.0570 (6)	
H4	0.5000	0.2500	0.2567	0.068*	
C5	0.58183 (14)	0.34178 (15)	0.00845 (6)	0.0401 (4)	
C6	0.56001 (18)	0.30343 (18)	−0.05269 (6)	0.0504 (4)	
H6A	0.6457	0.2621	−0.0685	0.060*	
H6B	0.5346	0.3868	−0.0746	0.060*	
C7	0.25630 (16)	0.36549 (17)	0.09927 (6)	0.0495 (4)	
H7A	0.1918	0.2897	0.0902	0.059*	
H7B	0.2873	0.4108	0.0651	0.059*	
H7C	0.2090	0.4338	0.1228	0.059*	
N1	0.5000	0.2500	0.04113 (6)	0.0335 (4)	
O1	0.65642 (13)	0.43332 (13)	0.02778 (5)	0.0606 (4)	

### Atomic displacement parameters (Å^2^)

**Table:**

	*U*^11^	*U*^22^	*U*^33^	*U*^12^	*U*^13^	*U*^23^
C1	0.0383 (9)	0.0299 (8)	0.0292 (9)	−0.0041 (6)	0.000	0.000
C2	0.0424 (7)	0.0318 (7)	0.0384 (8)	−0.0004 (5)	0.0050 (5)	0.0021 (5)
C3	0.0661 (10)	0.0451 (8)	0.0384 (8)	0.0064 (7)	0.0143 (7)	−0.0008 (6)
C4	0.0886 (17)	0.0525 (12)	0.0299 (10)	0.0066 (11)	0.000	0.000
C5	0.0418 (7)	0.0430 (7)	0.0355 (7)	−0.0079 (5)	0.0002 (5)	0.0049 (5)
C6	0.0596 (9)	0.0590 (9)	0.0324 (8)	−0.0113 (7)	0.0035 (6)	0.0031 (6)
C7	0.0411 (8)	0.0509 (8)	0.0564 (9)	0.0065 (6)	0.0056 (6)	0.0070 (7)
N1	0.0345 (8)	0.0372 (8)	0.0290 (8)	−0.0047 (6)	0.000	0.000
O1	0.0700 (8)	0.0646 (8)	0.0470 (7)	−0.0348 (6)	−0.0061 (5)	0.0056 (5)

### Geometric parameters (Å, °)

**Table:**

C1—C2^i^	1.3978 (15)	C5—N1	1.3916 (15)
C1—C2	1.3978 (15)	C5—C6	1.5064 (19)
C1—N1	1.431 (2)	C6—C6^i^	1.511 (3)
C2—C3	1.387 (2)	C6—H6A	0.9700
C2—C7	1.5008 (19)	C6—H6B	0.9700
C3—C4	1.3807 (19)	C7—H7A	0.9600
C3—H3	0.9300	C7—H7B	0.9600
C4—C3^i^	1.3807 (19)	C7—H7C	0.9600
C4—H4	0.9300	N1—C5^i^	1.3916 (15)
C5—O1	1.2012 (17)		
			
C2^i^—C1—C2	122.29 (17)	C5—C6—C6^i^	105.13 (8)
C2^i^—C1—N1	118.85 (8)	C5—C6—H6A	110.7
C2—C1—N1	118.85 (8)	C6^i^—C6—H6A	110.7
C3—C2—C1	117.83 (13)	C5—C6—H6B	110.7
C3—C2—C7	120.07 (12)	C6^i^—C6—H6B	110.7
C1—C2—C7	122.10 (13)	H6A—C6—H6B	108.8
C4—C3—C2	120.71 (14)	C2—C7—H7A	109.5
C4—C3—H3	119.6	C2—C7—H7B	109.5
C2—C3—H3	119.6	H7A—C7—H7B	109.5
C3—C4—C3^i^	120.63 (19)	C2—C7—H7C	109.5
C3—C4—H4	119.7	H7A—C7—H7C	109.5
C3^i^—C4—H4	119.7	H7B—C7—H7C	109.5
O1—C5—N1	123.75 (13)	C5—N1—C5^i^	112.40 (15)
O1—C5—C6	128.14 (13)	C5—N1—C1	123.80 (8)
N1—C5—C6	108.11 (12)	C5^i^—N1—C1	123.80 (8)
			
C2^i^—C1—C2—C3	0.13 (9)	O1—C5—N1—C5^i^	177.17 (18)
N1—C1—C2—C3	−179.87 (9)	C6—C5—N1—C5^i^	−3.48 (8)
C2^i^—C1—C2—C7	−179.44 (14)	O1—C5—N1—C1	−2.83 (18)
N1—C1—C2—C7	0.56 (14)	C6—C5—N1—C1	176.52 (8)
C1—C2—C3—C4	−0.27 (19)	C2^i^—C1—N1—C5	−73.92 (9)
C7—C2—C3—C4	179.31 (11)	C2—C1—N1—C5	106.08 (9)
C2—C3—C4—C3^i^	0.14 (10)	C2^i^—C1—N1—C5^i^	106.08 (9)
O1—C5—C6—C6^i^	−171.85 (18)	C2—C1—N1—C5^i^	−73.92 (9)
N1—C5—C6—C6^i^	8.8 (2)		

Symmetry codes: (i) −*x*+1, −*y*+1/2, *z*.
